# Supplementary material for: Interlaboratory Validation of Toxicity Testing Using the Duckweed Lemna minor Root-Regrowth Test
Source: Biology (Basel). 2021 Dec 27;11(1):37. doi: 10.3390/biology11010037 (PMC8772783; doi:10.3390/biology11010037)
Supplement: Supplementary file 1 [file biology-11-00037-s001.zip › biology-1455448-supplementary.pdf]

## Supplementary table

**Table S1. International standards (ISO, OECD, ASTM and US EPA) for aquatic bioassays.**

| Hydroecology \ International standards | Biotic factors | ISO | OECD | ASTM | US EPA | Total |
|---|---|---|---|---|---|---|
| Fresh (water, sediment) | Producer | 6 | 4 | 5 | 1 | 16 |
| | Consumer | 20 | 20 | 15 | 9 | 64 |
| | Decomposer | 7 | 3 | 1 | 0 | 11 |
| | Sub-total | 33 | 27 | 21 | 10 | 91 |
| Marine (water, sediment) | Producer | 2 | 0 | 2 | 1 | 5 |
| | Consumer | 5 | 1 | 11 | 9 | 26 |
| | Decomposer | 2 | 0 | 0 | 0 | 2 |
| | Sub-total | 9 | 1 | 13 | 10 | 33 |
| Total | | 42 | 28 | 34 | 20 | 124 |
